# Effect of Denture Disinfectants on the Mechanical Performance of 3D-Printed Denture Base Materials

**DOI:** 10.3390/polym15051175

**Published:** 2023-02-26

**Authors:** Nora S. Alkaltham, Reem A. Aldhafiri, Ahmad M. Al-Thobity, Hassan Alramadan, Hussain Aljubran, Ijlal Shahrukh Ateeq, Soban Q. Khan, Sultan Akhtar, Mohammed M. Gad

**Affiliations:** 1 Department of Substitutive Dental Sciences, College of Dentistry, Imam Abdulrahman Bin Faisal University, Dammam 31441, Saudi Arabia; 2 College of Dentistry, Imam Abdulrahman Bin Faisal University, Dammam 31441, Saudi Arabia; 3 Biomedical Engineering Department, College of Engineering, Imam Abdulrahman Bin Faisal University, Dammam 31441, Saudi Arabia; 4 Department of Dental Education, College of Dentistry, Imam Abdulrahman Bin Faisal University, Dammam 31441, Saudi Arabia; 5 Department of Biophysics, Institute for Research and Medical Consultations, Imam Abdulrahman Bin Faisal University, Dammam 31441, Saudi Arabia

**Keywords:** 3D printing, denture cleansers, mechanical testing, PMMA

## Abstract

Denture care and maintenance are necessary for both denture longevity and underlying tissue health. However, the effects of disinfectants on the strength of 3D-printed denture base resins are unclear. Herein, distilled water (DW), effervescent tablet, and sodium hypochlorite (NaOCl) immersion solutions were used to investigate the flexural properties and hardness of two 3D-printed resins (NextDent and FormLabs) compared with a heat-polymerized resin. The flexural strength and elastic modulus were investigated using the three-point bending test and Vickers hardness test before (baseline) immersion and 180 days after immersion. The data were analyzed using ANOVA and Tukey’s post hoc test (α = 0.05), and further verified by using electron microscopy and infrared spectroscopy. The flexural strength of all the materials decreased after solution immersion (*p* < 0.001). The effervescent tablet and NaOCl immersion reduced the flexural strength (*p* < 0.001), with the lowest values recorded with the NaOCl immersion. The elastic modulus did not significantly differ between the baseline and after the DW immersion (*p* > 0.05), but significantly decreased after the effervescent tablet and NaOCl immersion (*p* < 0.001). The hardness significantly decreased after immersion in all the solutions (*p* < 0.001). The immersion of the heat-polymerized and 3D-printed resins in the DW and disinfectant solutions decreased the flexural properties and hardness.

## 1. Introduction

Complete dentures (CDs) have been used for many decades to replace the functions and aesthetics in edentulous patients [1]. The main components of CDs include denture teeth and denture base materials. Most researchers have investigated the properties of denture base materials. Owing to its various satisfactory outcomes, such as its mechanical, physical, and aesthetic effects and cost-effectiveness, polymethyl methacrylate (PMMA) is considered to be the most common material used to fabricate conventional CDs [2]. However, its fracture susceptibility, tissue irritability, and denture-related infections are considered the main challenges for this type of material [3].

Denture base production through digital technologies, such as computer-aided design/computer-aided manufacturing (CAD/CAM) and 3D printing, has recently become possible owing to the advances in science and technology [4]. A reduced manufacturing time, an excellent precision, fewer patient visits, and enhanced patient comfort are all advantages of CAD/CAM-fabricated prostheses [5,6]. Stereolithography is usually the technology used for producing 3D-printed dentures, which involves the layer-by-layer laser beam scanning and polymerization of a methacrylate-based photocurable resin [5]. Meanwhile, 3D-printed technology (an additive method) has advantages over the subtractive method in terms of cost-effectiveness, resin material waste, and milling bur deterioration [5,6].

Dentures are a favorable environment for bacterial and fungal pathogens, such as *Streptococci* and *Candida* [7]. Denture stomatitis is caused by plaque and inadequate denture hygiene [8,9]. Additionally, the microorganisms adhering to dentures can cause halitosis, pneumonia, bacterial endocarditis, chronic pulmonary obstruction, and gastrointestinal infections [10]. Thus, the goal of denture care and maintenance is to remove the plaque biofilms forming on the surface of dentures, thereby preventing oral disease [11].

Dentures can be disinfected via different methods, including soaking in chemical solutions, brushing mechanically with a toothpaste, or using microwave radiation. These denture-cleansing techniques can clean the denture surfaces from stains, debris, and biofilms [12,13]. However, chemical cleaners have been reported to yield adverse effects on the properties of different prostheses [14,15,16,17,18]. The effects of different chemical disinfectants on different types of polymers have been widely studied. Despite their beneficial antimicrobial effect, some types of chemical disinfectants have been shown to deteriorate the mechanical properties of polymer-based materials [19]. A significant increase in surface roughness and a decrease in flexural strength have been reported when polymer-based materials have been subjected to some chemical disinfectants [19,20,21].

Several chemical denture-cleansing agents with antimicrobial properties have previously been investigated [12,22,23]. Among these agents, alkaline peroxides and alkaline hypochlorites are widely recognized [22]. Sodium hypochlorite (NaOCl) has been used in different concentrations and has shown highly antimicrobial effects [24,25]. However, an apparent adverse effect on the mechanical and physical properties of denture base resins has been observed. Peroxide-based effervescent solutions have shown favorable antimicrobial activity [26]. Effervescent tablets have been investigated in comparison with NaOCl and have shown a comparable antimicrobial effect, with minimal influence on the physical and mechanical properties of the denture base resins [22,24,25].

Since 3D-printed denture base resins have been recently introduced into clinical use, they have been subjected to denture care solutions and chemicals that may affect their strength. To the authors’ knowledge, no previous studies have investigated the effects of different disinfectant solutions on the mechanical behavior of 3D-printed resins. Therefore, this study was designed to investigate the effects of different immersion solutions on the flexural properties and hardness of the 3D-printed resins used for denture base fabrications. The null hypothesis was that the flexural strength, elastic modulus, and hardness of the 3D-printed denture base resins would remain unaffected when subjected to the different denture disinfectant solutions. Since the chemical structure and surface topography are important parameters when defining the mechanical performance of 3D-printed denture base materials, the specimens were evaluated using Fourier transform infrared spectroscopy (FTIR) and scanning electron microscopy (SEM).

## 2. Materials and Methods

### 2.1. Specimen Preparation

The sample size was calculated as previously described [23,24]. A total of 120 (40/material, 10/solution) acrylic resin specimens were required. The specimens were fabricated using three different materials: one heat-polymerized (HP) acrylic resin and two different 3D-printed resins (NextDent [ND] and FormLabs [FL]). Following ISO 20795-1:2013 standards [27], the specimens with a dimension of 64 × 10 × 3.3 ± 0.2 mm were prepared for the bending strength test. Meanwhile, after the fracture test, 10 fracture pieces were selected and used for the hardness test. The materials used for the specimen fabrication and procedure details are presented in Table 1.

For the 3D-printed specimens, the required dimensions were designed using the AutoCAD software and transferred STL files. The STL files were sent to the software of both NextDent 5100 (3D Systems, Vertex Dental B.V., Soesterberg, The Netherlands) and FL (FormLabs, Somerville, MA, USA). The specimens were printed following the manufacturer’s recommendations and the specified parameters seen in Table 1. After printing, the specimens were immersed in an alcohol bath (Isopropyl Alcohol 99.9%, Saudi Pharmaceutical Industries, Riyadh, KSA) for cleaning, followed by the post-curing process (Table 1). The supporting structures were removed after curing, and the specimens were then polished and stored in distilled water (DW) at 37 **°**C for 48 h.

### 2.2. Immersion Solutions and Immersion Protocol

A total of thirty specimens (10/material) were kept for testing without immersion. The immersion solution, preparation, and immersion protocols are presented in Table 2. The specimens were kept submerged in the respective solutions for 180 days (simulated) [28]. The resin specimens were taken out and washed thoroughly with running water, and later stored at room temperature in DW. In total, one investigator was assigned to perform the immersion procedures, wherein fresh solutions were prepared daily. The testing procedures were conducted for both the un-immersed and immersed specimens [23,29,30].

### 2.3. Testing Procedures

#### 2.3.1. Flexural Properties

The bending test was conducted following ISO recommendations and previously described procedures [27,31,32]. After their removal from water, each specimen was set on two vertical supports, 50 mm apart. The load was delivered to the specimen’s center at a 5 mm/min crosshead speed until the fracture. The fracture force (newtons) was recorded to calculate the flexural strength and elastic modulus [27,31,32,33].

#### 2.3.2. Hardness

The Vickers hardness test (Wilson Hardness; ITW Test & Measurement GmbH, Shanghai, China) was used to determine the hardness of the materials. Each specimen was placed on the testing machine, and three readings per specimen were recorded at different locations. A Vickers diamond indenter with a 25 gf load was applied for each 30 s. Each specimen’s final hardness value was calculated arithmetically by taking the average of the three readings [23,34].

### 2.4. FTIR Characterization

After treatment with the disinfectants (DW, effervescent tablets, and NaOCl), the chemical bonding of the three groups of specimens (HP resin, ND, and FL) was analyzed using FTIR (Nicolet 6700, FTIR spectrometer). The baseline data of all the specimens were also added for comparison. For the FTIR data, the spectra were recorded between an IR range of 4000 and 500 cm*^−^*^1^.

### 2.5. SEM Characterization

The effects of the different disinfectants on the three resin specimens were analyzed using a scanning electron microscope (FEI Company Inspect S50, Kohoutovice, Czech Republic). For this purpose, the fractured surfaces of the specimens were examined under the scanning electron microscope, operated at 30 kV as an accelerating voltage. The SEM specimens were gold-coated to minimize the surface charging effect, owing to the non-conductive nature of the polymeric resins. SEM images were taken in the secondary electron image mode; the WD was 10 mm, and the representative magnification for all the specimens was ×1000.

### 2.6. Statistical Analysis

The means and standard deviations (SDs) were computed as a part of the descriptive data analysis. The Shapiro–Wilk test was applied to check the data normality, and insignificant results showed normally distributed data. For the inferential data, parametric tests were performed to study the variation of the properties in the different groups; a one-way ANOVA was executed. A pairwise comparison between the groups was conducted by applying Tukey’s post hoc test. A two-way ANOVA was executed to study the combined impact of the disinfectants and materials on the tested properties. All *p*-values of <0.05 were considered statistically significant.

## 3. Results

The means, SDs, and significant differences between the tested groups for all the investigated properties are summarized in Table 3. The flexural strength of all the materials decreased after their immersion in the solutions when compared with the baseline (*p* < 0.001). In the analysis of the effects of the denture cleansers (DW and NaOCl) per material (HP resin, ND, and FL), both cleansers significantly reduced the flexural strength (*p* < 0.001), with the lowest values recorded with the use of NaOCl for all the materials. In the comparison between the materials per immersion solution, ND and FL showed similar behaviors in their decreasing of the flexural strength compared with the HP resin. Meanwhile, there was no significant difference between ND and FL per immersion solution (*p* > 0.05).

The elastic modulus of all the materials showed no significant difference between the baseline and after the DW immersion (*p* > 0.05). Meanwhile, the effervescent tablet and NaOCl immersion significantly decreased the elastic modulus of all the materials (*p* < 0.001), with the lowest value recorded after the NaOCl immersion (Table 3). In the comparison between the materials per immersion solution, for all immersion solutions, ND and FL significantly decreased the elastic modulus compared with the HP resin. No significant differences were noted between ND and FL (*p* > 0.05) when the materials were immersed in DW and effervescent tablets, while the NaOCl immersion significantly decreased the elastic modulus (*p* < 0.001). The FL immersed in NaOCl showed the lowest elastic modulus (Table 3).

In the comparison of the hardness of the tested materials before and after their immersion in the different solutions, the hardness was found to significantly decrease after immersion (*p* < 0.001), except for ND and FL after the DW immersion, which presented no significant difference with the baseline (*p* > 0.05). Based on the solution, ND and FL significantly decreased the hardness compared with the HP resin; there was no significant difference between ND and FL. The lowest hardness values were recorded after the NaOCl immersion, and in ND (Table 3).

Table 4 summarizes the two-way ANOVA results of all the tested properties. The combined effect of the two independent variables (the disinfectant and denture base material) on the flexural strength, elastic modulus, and hardness was tested. The analysis revealed that the combined effect of both independent variables was significant for each tested property (*p* < 0.001).

**Figure 1:**
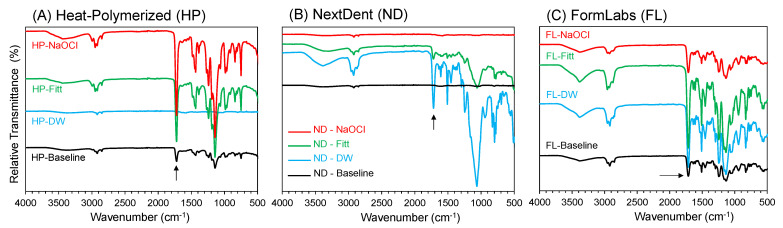
FTIR spectra of the three groups of specimens disinfected with DW, effervescent tablets, and NaOCl. (**A**) HP resin, (**B**) ND, and (**C**) FL. The baseline FTIR spectrum of the specimens is also shown. Fitt, effervescent tablets.

The effects of the disinfectants on the chemical nature of the HP resin, ND, and FL specimens were analyzed using FTIR. The surface topography of the fractured specimens was also evaluated using SEM to further highlight the mechanical behaviors. The FTIR results for the HP resin, ND, and FL specimens disinfected with the DW, effervescent tablets, and NaOCl are shown in Figure 1. The spectra of all the specimens showed the characteristic bands (e.g., carbonyl and methyl groups at 1722–1144 cm^−1^) of the polymeric resins [35,36]. However, there was an apparent difference between the baseline spectra and the spectra after the disinfection of all the specimens. In particular, the baseline spectrum of ND showed the characteristic bands with low intensities (Figure 1B). The main band appeared at approximately 1705 cm^−1^ (carbonyl groups in PMMA chains), which was stronger for the HP resin and FL specimens than for the ND specimen, as highlighted by the black arrows. A similar trend was found for another important band appearing at approximately 1060 cm^−1^, which was attributed to C-O-C stretching vibrations, wherein the HP resin and FL showed intense bands.

**Figure 2:**
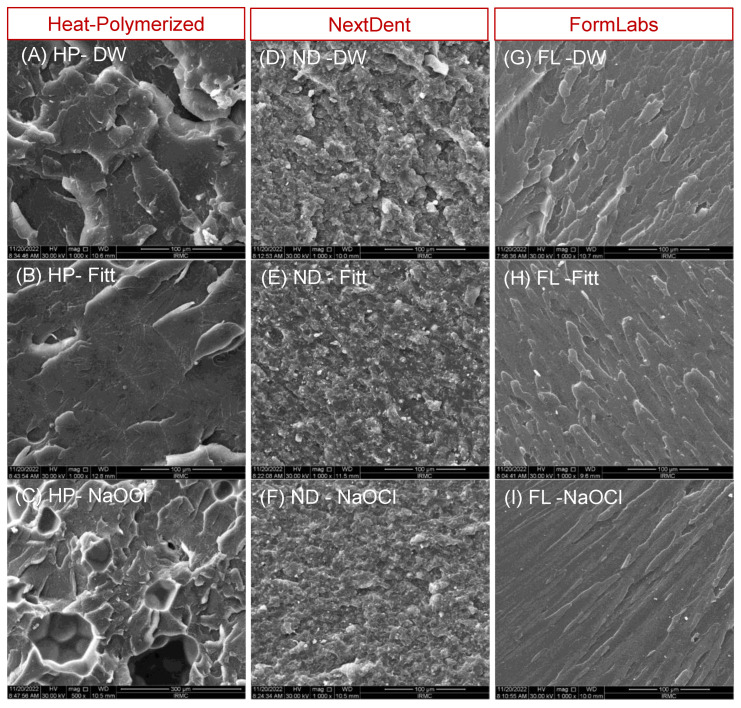
Representative SEM images of the three fractured materials (HP resin, ND, and FL) disinfected with the three solutions (DW, Fitt, and NaOCl). (**A**) HP–DW, (**B**) HP–Fitt, (**C**) HP–NaOCl, (**D**) ND–DW, (**E**) ND–Fitt, (**F**) ND–NaOCl, (**G**) FL–DW, (**H**) FL–Fitt, and (**I**) FL–NaOCl. SEM magnification for all specimens: ×1000, scale bar: 100 µm, WD: approximately 10 mm. Fitt, effervescent tablets.

The SEM analysis of the fractured specimens depended mainly on the surface features (i.e., the surface roughness, particulates, and surface irregularities). For example, a material with high strength needed more energy for its failure; hence, it showed more surface roughness and irregularities. This kind of failure was categorized as a ductile fracture. Meanwhile, a material with low strength was fractured with a smooth background and categorized as a brittle fracture. Figure 2 shows the representative SEM images of the three materials disinfected with the three disinfectants. The HP resin immersed in the DW (Figure 2A) showed rough surface features with more and sharp lamellae, representing a ductile fracture. These features changed from sharp lamellae to faint lamellae with a smooth background after the immersion in effervescent tablets (Figure 2B), and extended to voids and a patch-like appearance after the immersion in NaOCl (Figure 2C). For the 3D-printed resins (Figure 2D–F), the features were different, especially for ND, wherein some cracks and small, faint, irregular lamellae were observed after the immersion in DW and effervescent tablets; these features decreased with void formations after the immersion in NaOCl. Meanwhile, FL showed a uniform lamellar distribution in DW (Figure 2G); this feature decreased gradually (Figure 2H) to a slightly lamella-like appearance with a smooth background (Figure 2I) after the immersion in effervescent tablets.

## 4. Discussion

This study was conducted to investigate the effects of immersion solutions (DW, effervescent tablets, and NaOCl) on the mechanical performance of one HP resin and two 3D-printed resins (ND and FL). The null hypothesis was rejected, as all the disinfectant solutions had a significant impact on the tested properties, such as the flexural strength, elastic modulus, and hardness of the 3D-printed resins used for denture base fabrication.

In the present study, the HP resin contained a cross-linking agent (ethylene glycol dimethacrylate), forming a cross-linked polymer, which restricted the chain movement when stressed and subsequently improved the mechanical properties [37]. Meanwhile, the 3D-printed resins were composed of a monomer and an oligomer, such as bisphenol A-glycidil dimethacrylate or urethane dimethacrylate, which were subjected to ultraviolet light for polymerization and resulted in a cross-linked polymer [38]. However, an uncured monomer requires a post-curing process to cross-link the unreacted monomers and to complete the polymerization of the printed specimens, which subsequently provides the specimens with a high mechanical performance [39,40,41]. The present study showed a decreased flexural strength of the 3D-printed resins compared with that of the HP resin, which agrees with previous reports [4,27,32,39,42]. This reduction may be attributed to the printing nature and polymerization technique [4,32]. Furthermore, the 3D-printed specimens were printed layer by layer, followed by photo-polymerization until they were completely printed in the required dimension. The weak interlayering bonding and double-bonding of the resin matrix may be attributed to the unreacted monomer [32], in addition to the photo-polymerization method with a low degree of conversion and more residual monomers, even with the recommended post-polymerization process [4,39,43].

Among the factors that affect the strength of 3D-printed resins is their aging and storage in water, as well as their immersion in chemicals [44]. Denture cleansers are prepared and dissolved in water, making water their most abundant component [45]. Owing to the nature and polarity of resin materials, the absorbance of water molecules easily occurs, allowing for the easy penetration of the resin network and the diffusion of monomers and/or additives from the resin network [46]. The absorbed water displaces the polymer chains, penetrates the polymer network, and reduces the intermolecular force that creates internal stress, resulting in weak mechanical properties [42,45]. There are two factors affect the amount of absorbed water and the leaching out of monomers: the coefficient of the water diffusion of resins and the amount of residual monomers. The first factor is related to the time needed for material saturation with water, while the second factor affects the amount of absorbed water that replaces the leached out residual monomer [45,47]. In previous studies, polymers have been subjected to chemical degradation when immersed in aqueous solutions via two ways: hydrolysis and enzymatic reaction; the reaction starts with a side chain attack, resulting in by-products and network property deteriorations [45]. Additionally, the amount of absorbed water plays a role in resin degradation, as water molecules penetrate and fill the spaces between the polymer chains, pushing them apart and forming a secondary weak force (van der Waals) between the polymeric chains, finally resulting in resin expansion and swelling [48], and mechanical performance weakening. This process is exaggerated with time, as water molecules act as a plasticizer, affecting the mechanical behaviors of the resins [29,42,45,46]. This confirmed the current findings: after the immersion of all the resins in DW, the flexural strength decreased significantly compared with the baseline. This decrease may be attributed to the water sorption effect of the HP and 3D-printed resins, as reported previously [49]. Meanwhile, there was no difference in the strength between the 3D-printed resins, and ND presented the lowest flexural strength (69.8 ± 2.4 MPa), but this is still higher than the ISO recommendations and suitable for clinical use [50].

Herein, an apparent decrease in the flexural strength was recorded when both the 3D-printed resins were immersed in the effervescent tablets and NaOCl. This finding may be related to the poor strength of the 3D-printed resins, which was exaggerated after immersion, owing to the chemical composition of each disinfectant [24]. Denture cleansers have oxide-releasing agents and enzymes, which may cause the expansion of intermolecular spaces, aiding in the leaching out of degraded network contents and the penetration of the water and chemicals present in denture-cleansing solutions [51]. During immersion in denture cleansers, the water molecules associated with the chemicals gradually permeate from the surface into the resin matrix [52]. This prolonged immersion could result in an irreversible deformation and surface expansion, inducing various stresses and osmotic pressures [52,53]. Additionally, the water absorption rate of the 3D-printed resins is higher than that of the HP resins [49], which might be another explanation for the decreased flexural strength of the 3D-printed resins after their disinfection in the current study. This finding contradicts previous reports [14,54,55] that there is no significant reduction in the flexural strength of denture base resins when they are immersed in effervescent tablets. This variation in the results may be attributed to the differences in the immersion time, duration, and the brand of resins used.

NaOCl immersion significantly reduced the flexural strength of the tested resins, and the 3D-printed resins showed values lower than the ISO recommendations. Similarly, Davi et al. [56] and Kurt et al. [57] demonstrated that 1% NaOCl immersion significantly decreased the flexural strength of the materials. This decrease is linked with the sorption of the NaOCl aqueous solution and its active chlorine content. The absorbed solution acts as a plasticizer, in addition to the possibility of it altering the chemical structure [58]. Furthermore, the residual monomer solubility increased, owing to its active chlorine content, which led to more leachability of the residual monomer, compensated by more water sorption, which is considered to be the main factor that affects denture base resin strength [15]. Other studies have explained this decrease as based on the fact that denture cleansers interact with PMMA denture base resins and influence the surface integrity of the polymer resin chains [16,54,59]. Several studies have reported a decrease in the flexural strength with 1% NaOCl [56,59].

The effects of the disinfectant solutions were similar for both ND and FL, with no significant difference according to the solution. The NaOCl immersion significantly decreased the flexural strength compared with the effervescent tablet immersion. This finding may be attributed to the different chemical compositions of each disinfectant [14,24]. A previous study has compared the flexural strength of denture base resins after immersion in effervescent tablets for 30 min and in 1% NaOCl for 10 min; the flexural strength decreased when the specimens were immersed in NaOCl [14], consistent with the present findings.

The rigidity or the flexibility of resins is expressed as the elastic modulus, wherein a higher elastic modulus indicates a rigid material [4,60]. Denture base materials should have some rigidity to tolerate the stress and be flexible to certain levels to distribute the load equally and decrease the fracture risk [61]. Herein, the elastic modulus of the resins mimicked the flexure strength, wherein the elastic modulus of the HP resin was higher than that of the 3D-printed resins. Similarly, Fouda et al. [27] compared the elastic modulus between the 3D-printed and HP resins and reported a low elastic modulus of the 3D-printed resins. In the study by Fouda et al. [27], the elastic modulus was higher than the ISO recommendations (2000 MPa) [50]; in the present study, only the baseline elastic modulus of the HP resin was close to the ISO recommendations, while the values of all the other HP and 3D-printed resins were lower. This finding may be attributed to the different immersion solutions used: Fouda et al. [27] tested all the specimens without aging. The elastic modulus of the HP and 3D-printed resins decreased with effervescent tablets but sharply declined with NaOCl. This finding may be attributed to the water and chemical uptake when immersed in these disinfectant solutions. No studies have evaluated the effects of denture cleansers on the elastic modulus of the 3D-printed resins, making comparisons with previous studies inappropriate. However, the low modulus could similarly be explained as the flexural strength, since both were tested under the same load, direction, and condition, and expressed as flexural properties [27,39].

The hardness of the tested resins decreased when immersed in the effervescent tablets and NaOCl, with the lowest values recorded with the NaOCl. The absorbed fluids can penetrate the interpolymeric chains, affecting the bonds, and act as a plasticizer, decreasing the strength [61]. This decrease may be explained by the chemical ingredients and the plasticizer action of the absorbed solution, causing material deformation [25,29]. The absorbed solution intervenes with the polymer chains, leading to resin network swelling and changes in the chemical structure of the polymer matrix, with minimal softening of the resin [62]. The present findings agree with the report by Alqanas et al. [25] that two effervescent denture-cleansing solutions significantly decrease the hardness of 3D-printed denture base resins. Additionally, Atalay et al. [29] and Kurt et al. [57] reported that NaOCl reduced the hardness of CAD/CAM [29] and HP denture base resins [57].

Denture care is important for denture longevity and patient satisfaction. Thus, the selection of appropriate denture bases and cleansers is crucial. Although there were no substantial differences in the investigated properties between the 3D-printed resins, the strength was lower than the ISO recommendations when immersed in denture disinfectants. Both denture cleansers adversely affected the strength of the tested materials. This finding was confirmed through the analysis of the combined effects of the types of resins and disinfectants. Therefore, the types of resins and disinfectants should be considered in denture hygiene evaluation.

Denture home care using disinfectants might influence the physical properties and mechanical behaviors of denture base resins, regardless of their fabrication methods [63]. A balance between the disinfectants’ effectiveness and their effects on the resin properties is required. Therefore, the strength of new resins for denture fabrication using new technology has become a research focus. Considering the decreased strength of the 3D-printed resins after their immersion in the selected disinfectants in our study, the disinfectants are not recommended for 3D-printed resins. Finally, the types of denture disinfectants and base resins must be considered in the development of a prosthetic device. The disinfectants must be effective without being detrimental to the materials of which the prosthesis is made.

The current findings should be interpreted with caution, owing to the limitations related to in vitro study conditions. One of these limitations is that only two 3D-printed resins and two disinfectants were used. Furthermore, the specimens were tested in conditions differing from oral conditions, such as the absence of oral fluids, dietary intake, and occlusal stress. In addition, flat specimens do not simulate actual denture configurations. The immersion time simulating 180 clinical uses indicates the need for further investigations into long-term clinical use with different disinfectants. Moreover, future investigations into different resin brands in conditions that mimic the oral environment are recommended.

## 5. Conclusions

1. The 3D-printed resins displayed a lower flexural strength, elastic modulus, and hardness than the HP acrylic resin did.
2. The immersion of the HP and 3D-printed resins in DW and the disinfectant solutions decreased their flexural properties and hardness.
3. Effervescent tablet and NaOCl immersion had an adverse influence on the strength of the 3D-printed denture base resins, and the most adverse effect was found after the NaOCl immersion.
4. The types of disinfectant and denture base material affected the strength of the resins; therefore, the selection of appropriate types of material and disinfectant is crucial for denture longevity.

## Figures and Tables

**Table 1:** Materials used for specimen fabrication and procedure details.

	Materials		
	Heat-Polymerized Resin	NextDent	FormLabs
**Manufacturer**	Major.base.20, Major Prodotti Dentari SPA, Moncalieri, Italy	Denture 3D+, NextDent B.V., Soesterberg, The Netherlands	FormLabs Denture Base LP, FormLabs, Somerville, MA, USA
**Composition**	Powder: Polymer (PMMA) þ initiator (benzoyl peroxide) (0.5%) þ pigments (salts of cadmium or iron or organic dyes) Liquid: Monomer (MMA) þ cross-linking agent (ethylene glycol dimethacrylate 10%) þ inhibitor (hydroquinone)	Ethoxylated bisphenol A dimethacrylate 7,7,9 (or 7,9,9)-trimethyl-4,13-dioxo-3,14-dioxa-5,12- diazahexadecane-1,16-diyl bismethacrylate 2- hydroxyethyl methacrylate silicon dioxide diphenyl(2,4,6-trimethylbenzoyl)phosphine oxide titanium dioxide	55–75% *w*/*w* urethane dimethacrylate, 15–25% *w*/*w* methacrylate monomers, and <0.9% *w*/*w* phenyl bis(2,4,6-trimethylbenzoyl)-phosphine oxide
**Polymerization method**	Heat polymerization	3D printing with digital light processing	3D printing with stereolithography
**Fabrication procedure**	Wax specimens are invested within a metal flask. Wax elimination results in molds packed with dough acrylic resin. For polymerization, flasks are placed into a thermal curing unit (90 min at 74 °C and then 30 min at 100 °C).	(Photo polymerized) printed layer by layer (50 µm layer thickness) 0-degree printing orientations Post-curing machine (LC-D Print Box [3D systems]) and conditions (30 min/60 °C)	(Photo polymerized) printed layer by layer (50 µm layer thickness) 0-degree printing orientations Post-curing machine (FormCure) and conditions (30 min/60 °C)
**Polishing technique**	Specimens are cleaned and polished using an automated polishing machine (Metaserv 250 grinder-polisher; Buehler GmbH) with a mounted silicon carbide paper with different grits (800, 1500, and 2000).		

**Table 2:** Immersion solution preparation and immersion protocol.

Immersion Solution	Brand Name or Manufacturer	Composition	Preparation and Immersion Procedure
Distilled water	Distilled water	------	Immersion throughout the experiment at room temperature
Effervescent tablet (Fittydent alkaline peroxides)	Fittydent super (Fittydent International GmbH, Vienna, Austria)	Polyvinylpyrrolidone, sodium bicarbonate, sodium carbonate peroxyhydrate, potassium monopersulfate, sulfamic acid, sodium perborate monohydrate, sodium lauryl sulfate, tetraacetylethylenediamine, aroma, color C.I. 42090	One tablet dissolved in 200 mL warm tap water (40 °C) for 5 min Repeated 180 times
Sodium hypochlorite	Sodium hypochlorite solution	Sodium hypochlorite solution, 1% active chlorine	(a) Solution of 5.25% sodium hypochlorite (1:5 dilution) diluted to obtain 1% sodium hypochlorite by adding 50 mL sodium hypochlorite to 200 mL water (b) Immersion for 10 min at room temperature Repeated 180 times

**Table 3:** Means, SDs, and significant differences between the groups for all tested properties.

Tested Property	Group	Heat-Polymerized Resin	NextDent	FormLabs	*p*-Value
		Mean (SD)	Mean (SD)	Mean (SD)	
**Flexural strength** **(MPa)**	Baseline	95.9 (2.9)	73.2 (1.6) ^A^	74.0 (1.8) ^A^	<0.001 *
	DW	87.4 (1.9)	69.8 (2.4) ^A^	70.7 (2.2) ^A^	
	Effervescent tablets	74.9 (2.4)	66.0 (1.9) ^A^	65.2 (2.3) ^A^	
	NaOCl	69.7 (2.4)	62.1 (3.0) ^A^	61.8 (3.4) ^A^	
***p*-value**		<0.001 *	<0.001 *	<0.001 *	
**Elastic modulus** **(MPa)**	Baseline	1949.2 (70.7) ^a^	1215.0 (88.5) ^a,A^	1144.9 (67.1) ^a,A^	<0.001 *
	DW	1831.9 (96.1) ^a^	1191.9 (83.0) ^a,A^	1057.9 (73.9) ^a,A^	
	Effervescent tablets	1445.9 (225.4)	926.2 (64.3) ^A^	840.4 (86.7) ^A^	
	NaOCl	1110.6 (81.1)	741.3 (65.9)	567.2 (100.7)	
***p*-value**		<0.001 *	<0.001 *	<0.001 *	
**Hardness** **(VHN)**	Baseline	39.22 (3.04)	26.93 (2.32) ^a,A^	24.25 (2.07) ^a,A^	<0.001 *
	DW	35.10 (1.99)	22.72 (2.70) ^a,A^	22.47 (2.11) ^a,A^	
	Effervescent tablets	30.23 (2.81)	17.02 (2.26) ^A^	16.17 (3.37) ^A^	
	NaOCl	23.99 (2.47)	11.63 (2.21) ^A^	13.35 (1.67) ^A^	
***p*-value**		<0.001 *	0.003 *	0.007 *	

* Statistically significant at 0.05. ^a^ Statistically insignificant difference in each column; ^A^ statistically insignificant difference in each row; missing alphabets, significant difference. SD, standard deviation.

**Table 4:** Two-way ANOVA results of the tested properties.

	Source	Type III Sum of Squares	df	Mean Square	F	Sig.
**Flexural strength** **(MPa)**	Disinfectant	4729.777	3	1576.592	270.877	<0.001 *
	Material	5239.736	2	2619.868	450.124	<0.001 *
	Disinfectant × material	985.368	6	164.228	28.216	<0.001 *
	Error	628.595	108	5.820		
	Total	644,866.264	120			
**Elastic modulus** **(MPa)**	Disinfectant	7,479,885.467	3	2,493,295.156	244.371	<0.001 *
	Material	10,646,980.851	2	5,323,490.426	521.762	<0.001 *
	Disinfectant × material	432,700.169	6	72,116.695	7.068	<0.001 *
	Error	1,101,914.729	108	10,202.914		
	Total	183,520,026.088	120			
**Hardness (VHN)**	Disinfectant	3132.362	3	612.087	78.413	<0.001 *
	Material	235.249	2	231.301	31.846	<0.001 *
	Disinfectant × material	114.398	6	21.752	2.982	0.002 *
	Error	802.436	108	7.767		
	Total	65,109.883	120			

* Statistically significant difference.

## Data Availability

The data can be obtained from the corresponding author upon reasonable request.
